# Robotic optimization of powdered beverages leveraging computer vision and Bayesian optimization

**DOI:** 10.3389/frobt.2025.1603729

**Published:** 2025-06-09

**Authors:** Emilia Szymańska, Josie Hughes

**Affiliations:** ^1^ Department of Mechanical and Process Engineering (D-MAVT), Eidgenössische Technische Hochschule (ETH) Zürich, Zürich, Switzerland; ^2^ CREATE Lab, École Polytechnique Fédérale de Lausanne (EPFL), Lausanne, Switzerland

**Keywords:** Bayesian optimization, computer vision, robotics, food analysis, food optimization

## Abstract

The growing demand for innovative research in the food industry is driving the adoption of robots in large-scale experimentation, a shift that offers increased precision, repeatability, and efficiency in product manufacturing and evaluation. This paper addresses this need by introducing a robotic system that extends automation into optimization and closed-loop quality control, using powdered cappuccino preparation as a case study. By leveraging Bayesian Optimization and image analysis, the robot explores the parameter space to identify the ideal conditions for producing cappuccino with high foam quality. A computer vision-based feedback loop further improves the beverage by mimicking human-like corrections in preparation process. Findings demonstrate the effectiveness of robotic automation in achieving high repeatability and enabling extensive exploration of system parameters, paving the way for more advanced and reliable food product development.

## 1 Introduction

Food science is starting to play a significant role in the worldwide search for life improvements. Considering the growth of the human population and the dangers of human-induced pollution, the need to move towards more sustainable and efficient food sources is of utmost priority. To address this challenge, it is essential that these products are obtained in an optimized manner. Moreover, they must satisfy end-users’ sensory perceptions to ensure widespread acceptance and adoption (Kobayashi and Benassi, 2015; Kang et al., 2022). Research carried out in this sector requires high repeatability and accuracy in experiments whose main objective is to understand complex physical and chemical reactions occurring in food or drink preparation (Juriaanse, 2006; Tuorila and Monteleone, 2009; Buttriss, 2013).

Investigation of these aspects is typically achieved through manual lab experiments which can be slow and costly because of the extensive exploration of experimental parameters and conditions (Arteaga et al., 1994). Due to the challenges resulting from high stochasticity, automation is necessary to be applied to as many aspects as possible to reduce the influence of external factors on the preparation process. Additionally, the experimental food and drink preparation should mimic human behavior, which can be challenging as it requires both sensory and physical interactions with the product (Tuorila and Monteleone, 2009). One plausible solution for automating such scientific experiments is offered by a combination of robotics with computer vision and optimization (Duong et al., 2020), allowing for precise repetition, human behavior simulation, and intelligent data capture and analysis (Khan et al., 2018; Iqbal et al., 2017).

Research exploring robots’ utility in a kitchen still faces a multitude of challenges, especially in the area of sensory perception. In the systems employing robots to analyze and optimize food, a variety of evaluation solutions have been implemented–user feedback (Junge et al., 2020), salinity sensors (Sochacki et al., 2021a) or tactile assessment (Sochacki et al., 2021b; Scimeca et al., 2019). Whilst computer vision has been investigated for use in the food processing and food science industry (Ma et al., 2016; Külcü, 2018; Deotale et al., 2020), there has been limited exploration of the use of computer vision as a means of providing rapid feedback into the food optimization process. This could assist in enabling large scale, fully automated optimization of food products and their making processes with non-invasive and cheap sensing mechanisms.

**FIGURE 1:**
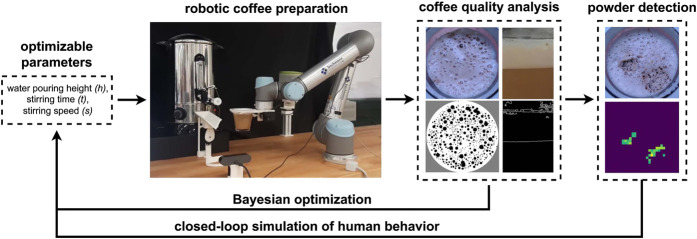
Robot Food Scientist. The robot setup with integrated computer vision is used to optimize the parameters of the beverage preparation, and to simulate human behavior in response to detection of undissolved powder clumps.

To address this goal, *Robot Food Scientist* is proposed–a robotic system which can automatically prepare beverages with various input parameters, evaluate their quality and optimize their creation, as presented in Figure 1. The selected case study is powdered cappuccino, with the foam being regarded as the primary quality indicator. Computer vision analysis of the foam is leveraged to simulate human responses to the visual characteristics of the beverage, with a particular emphasis on detecting and removing undissolved powder clumps in the closed-loop control system. Additionally, model-free optimization methods are used to find the optimal process for reconstitution. For optimization of foam-based beverages, Bayesian Optimization (BO) is proposed (Nguyen, 2019), as it is particularly suited for sequential analysis and global optimization of black-box functions without requiring assumptions on specific functional forms (Shahriari et al., 2015). The use of automation allowed for experiments with a high repeatability and also for much larger exploration of the different parameter combinations.

In summary, the paper makes the following contributions.

• It introduces the application of robotics and computer vision to conduct large-scale experiments in beverage preparation and quality analysis in an automated manner. A case study on powdered cappuccino demonstrates the effectiveness of this approach, with over a hundred coffees prepared and systematically evaluated.

• It defines a set of adjustable system parameters and meaningful quality metrics for foamed beverages and provides an analysis of the relationships between metrics.

• It develops a closed-loop control system allowing for the automated detection and removal of undissolved powder clumps, successfully mimicking human behavior in addressing product imperfections.

• It demonstrates that Bayesian Optimization is an effective method for identifying optimal preparation parameters that lead to the highest quality in consumable products.

## 2 Related works

### 2.1 Culinary robotics

Robots, as a means of addressing repetitive tasks, present a promising solution for food preparation automation. In the culinary domain, there has been a noticeable increase in research on applying robotic solutions for culinary scenarios (Beetz et al., 2011a; Danno et al., 2021; Bollini et al., 2011a; Beetz et al., 2011a; Bollini et al., 2011a; Satici A. et al., 2016; Ilic and Hughes, 2023). That includes such non-trivial tasks as correctly tossing a pizza dough (Satici A. C. et al., 2016), preparing a stir-fry dish (Liu et al., 2022), or making an omelet from scratch (Junge et al., 2020). Various approaches have been proposed for the implementation of these complex robotic systems. Techniques include demonstration learning (Schmitz et al., 2023), integrating Internet of Things into the system (Zhao et al., 2015), or employing Large Language Models to fulfill recipe instructions and monitor the state of food (Sakib and Sun, 2024; Kawaharazuka et al., 2024).

In this study, a fixed set of robot sequences is defined, and the execution of these sequences is enhanced using computer vision as a feedback mechanism from the environment. The selected case study involves the preparation of powdered cappuccino, where hot water is added while mixing to achieve reconstitution. This process results in the dissolution of the powder into the liquid and the creation of foam through aeration. To the best of current knowledge, this is the first application of robotics combined with computer vision for the preparation and analysis of a reconstituted beverage.

A notable challenge in the reconstitution process is the potential formation of undissolved powder clumps. In typical scenarios, a human operator would respond with additional mixing or a squishing motion to eliminate these clumps. Simulating this behavior in a robotic system is difficult, as it relies on visual detection of clumps, a phenomenon that does not occur consistently. An effective closed-loop control approach is introduced in this research to address this challenge, which, to the best of current knowledge, has not yet been explored in existing food industry research.

### 2.2 Foamed beverages analysis

An important area of focus in food optimization and process automation is the study of powder reconstitution, commonly used in products like coffee, soups, and other beverages (Fang et al., 2007). Specifically, the creation of foam is of high importance to both the drinks industry and consumers (Shweta et al., 2020; Schuchmann, 2007), with many studies focusing on the optimization and the understanding of the creation and formation of foams. Factors such as water temperature, amount of mixing, pour height, and vessel size all affect the aeration of the beverage and the reconstitution process (Labbe et al., 2021), thereby impacting the sensory preference among consumers (Deotale et al., 2020).

Computer vision has made it possible to automatically assess various quality indicators of foam, including its decay curve (Cimini et al., 2016), height (Gonzalez Viejo et al., 2016), or the distribution of bubble sizes (Gonzalez Viejo et al., 2016; Hendriks, 2020). Inspired by research in areas such as carbonated beverages (Viejo et al., 2019; Barker et al., 2002) and flotation froth analysis (Aldrich et al., 2022), where algorithms like watershed segmentation (Zhang et al., 2022), Hough transform (Romachev et al., 2020), and valley-edge detection (Aldrich et al., 2022) are used for bubble measurement, a specialized computer vision pipeline was developed. This pipeline incorporates three preprocessing approaches specifically designed to analyze bubbles in cappuccino foam. Additionally, a foam height measurement algorithm was created to investigate the relationship between foam height and bubble characteristics.

### 2.3 Experimental optimization of food properties

Robotics-driven food preparation optimization remains an under-explored area of research. The variations of Bayesian Optimization (BO) have found applications in food processing optimization (Junge et al., 2020; Banga et al., 2003). Its ability to efficiently explore complex parameter spaces is particularly well-suited for optimizing food processing tasks that involve multiple interacting variables. Tree-Structured Parzen Estimator (TPE) (Watanabe, 2023) is an alternative black-box method widely used in optimization tasks. However, due to its poor convergence in early experiments, BO was ultimately applied in the final optimization stage, yielding successful results as presented in this study. Specifically, this study explores how parameters such as the height of water pouring and the stirring dynamics affect the foam creation and the reconstitution process of powdered beverages. Optimization targets microfoam–a foam characterized by numerous small bubbles, which improves both the visual appeal and the mouthfeel of the beverage (Huppertz, 2010).

To conclude, despite the promising advances in literature, there remains a research gap in combining non-invasive image analysis techniques with a closed-loop robotic system for beverage quality evaluation and optimization. While various sensor and feedback methods have already been explored, the use of camera-only input for decision-making in food preparation is still underdeveloped. In particular, few studies demonstrate a fully automated pipeline for large-scale experiments, especially with the focus on parameter exploration and adaptive correction of beverage reconstitution based on visual feedback.

To address this gap, the objective of this study is to design a robotic system capable of preparing powdered cappuccino in an optimized manner. The system aims to test the applicability of Bayesian Optimization in identifying preparation parameters resulting in high-quality foam, and to implement a image-based feedback loop to correct the beverage imperfections through additional actions such as stirring.

## 3 Methods

In this section, the computer vision pipelines, optimization methods, and robotic setup employed to create beverages with varying parameters, evaluate foam quality, and detect undissolved powder are presented. The code implementation along with a detailed explanation of the hardware and software components can be found under the following Gitlab repository: https://gitlab.com/roboccino.

### 3.1 Coffee preparation setup

**FIGURE 2:**
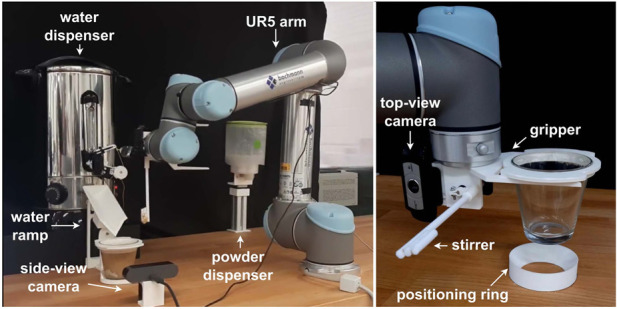
Experimental setup. The powder dispenser, water dispenser, water ramp and robot’s end effector were custom-designed and fabricated with the 3D printing technology.

Experimental analysis revealed that the parameters significantly influencing foam formation, while also being easily adjustable, are the height of water pouring (h) , mixing speed (s) , and mixing time (t) . The experimental setup that offers the variability of these parameters is shown in Figure 2. A 6 degree-of-freedom UR5 robot is equipped with a custom end effector which allows cups to be moved around. The end effector also features a DC-motor-controlled stirrer and a camera. Transparent cups make the drink easily visible, and a 3D printed rim has been added to the cups for easy and reliable movement. Furthermore, self-aligning cup holders have been designed to ensure cups are placed in a known location.

To dispense the powder, the cup is moved below a dispensing unit controlled by a stepper motor. The dispenser allows for a fixed quantity of the powder to be poured into the cup. The cup is then moved to the hot water dispenser, whose tap is operated by a servo controlling the duration of the open position period, thereby regulating the volume of added water. A ramp guides hot water from the dispenser to the cup, which allows for the pouring height to be varied with the use of a servo-driven cam mechanism. Water is added while the end effector’s stirrer mixes the content.

**FIGURE 3:**
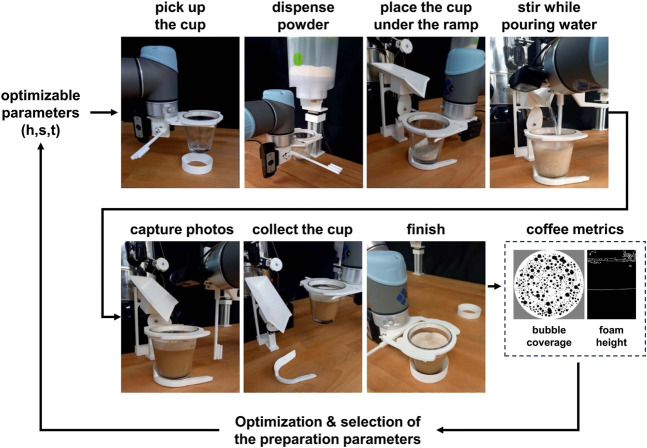
Open-loop coffee preparation steps. This procedure is executed in the optimal parameters search.

After mixing is finished, two cameras capture the state of the coffee: one overhead camera mounted on the end effector of the UR5, and the other one fixed on the table to capture the side view of the coffee. An anti-fog coating was applied to the overhead camera to prevent the coffee steam from affecting the image. Once the images are captured, they are analyzed with a computer vision pipeline, and the main controller makes a decision on to how to proceed with each experiment before the coffee is returned to its final location. The flowchart in Figure 3 summarizes the processes and the order of events that take place to make a single coffee.

The speed of mixing (s) corresponds to the speed of the stepper motor, with the range of possible speeds experimentally determined to be between s=[40%,100%] of the maximum stepper speed. The time of mixing t=[0s,60s] and the height of the water pouring h=[10cm,14cm] are also adjustable.

### 3.2 Coffee analysis

To assess the quality of the foam and detect the presence of undesired undissolved clumps of powder a number of computer-vision-based pipelines have been created. It is assumed that both the side view of the transparent cup and the top view of the foam are accessible.

#### 3.2.1 Foam bubbles

Bubble assessment is challenging due to the varying size of bubbles, their non-spherical shapes, and the reflective surface of the coffee. To create a robust bubble detection algorithm, three different detection pipelines leveraging blob detection are applied to the same image, and the results are then combined. Ideally, in the case of microfoam, no bubbles would be visible to this computer vision system.

**FIGURE 4:**
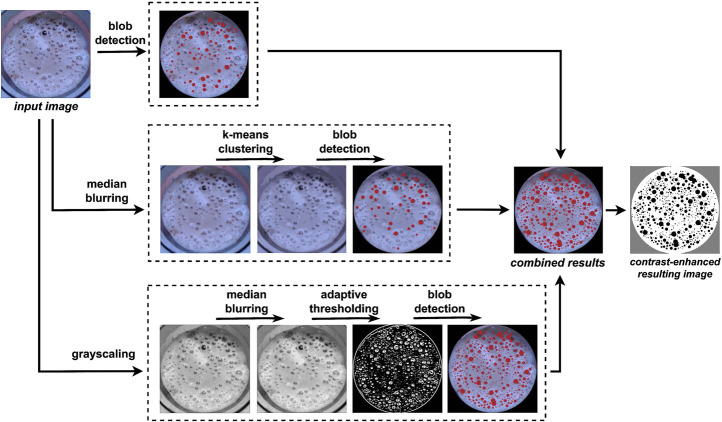
Bubble coverage determination pipeline. The results of three simultaneous processes are combined to identify the bubble coverage.

The first pipeline directly identifies small bubbles on the input image. The second one applies preprocessing with median blurring and K-means clustering to identify larger and non-spherical bubbles. The third detector uses grayscale conversion, median blurring, and adaptive thresholding, which is particularly effective for larger bubbles or those with reflections. The blobs detected with each of the three pipelines are then combined into an single black-and-white image, where the percentage area of blobs is determined by totaling the area of black pixels. This approach is summarized in Figure 4.

**FIGURE 5:**
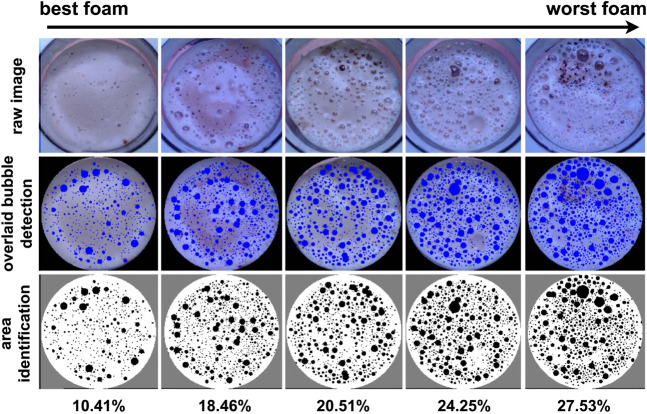
Bubble detection for foams of variable quality. This overview demonstrates that bubble coverage is an effective and reliable metric of the foam quality.

To demonstrate how this proposed method provides a representative metric for foam quality, coffees with foams of varying quality were prepared. As shown in Figure 5, the best foam has a very low area of bubbles (10.41%) , whereas the worst foam has an area of 27.53%. Within this range, the area metric increases monotonically with the decrease of the quality of the foam. This indicates that the metric corresponds to the visual quality of the foam and provides significant differentiation to capture the varying quality of foams.

#### 3.2.2 Foam height

**FIGURE 6:**
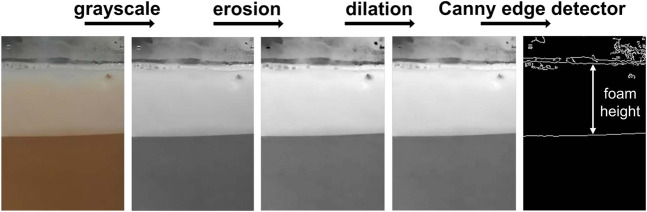
Foam height determination pipeline. The analysis is performed using a side-view image of the cup.

Foam height is a second metric used to define foam quality. Accurately assessing the foam height is challenging because, in the side view, the top of the foam can be difficult to see due to condensation on the glass and the presence of bubbles on the surface. To measure the foam height, the image is first converted to greyscale with an erosion and dilation applied, after which a Canny edge detector is used to identify the edges corresponding to the bottom and the top of the foam. The mean difference between these edges provides an estimation of the foam height. The approach is summarized in Figure 6.

#### 3.2.3 Clump detection

**FIGURE 7:**
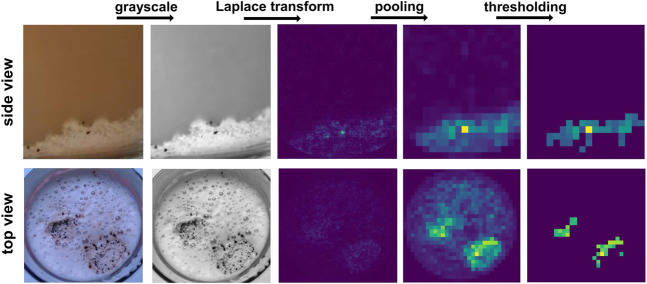
Clump detection pipeline. Undissolved powder clumps may be present both within the foam and at the bottom of the cup.

This detection focuses on the presence of undissolved powder clumps in both the foam and the bottom of the cup. A robust approach applicable for both of these cases has been developed. The images are first converted to greyscale, followed by an application of a Laplace transform. Then, a customized pooling returns a matrix filled with sums of absolute values of framed pixels. By thresholding pixel values, the presence and approximate area of the clumps can be determined. A demonstration of this approach is shown in Figure 7.

It is acknowledged that powder clumps forming a very thin layer at the bottom of the cup may remain undetected due to limited of visibility from the side. Inclusion of additional viewpoints, such as a bottom-facing camera, is considered for future work.

### 3.3 Closed-loop clump removal

To simulate consumer behavior in clump removal, the size of the clump (in pixels) is first detected (c) . Based on it, a proportional controller in the form of $t_{m}=\alpha c$ determines the additional mixing time $t_{m}$ required to remove the clump with the stirrer, either in the foam or at the bottom of the cup. The value of α was selected based on empirical tuning through informal testing, where various values were evaluated across representative scenarios. A value of 0.2 consistently presented the most favorable mixing performance, typically resulting in mixing times ranging from 5 to 20 s, depending on clump size.

### 3.4 Coffee optimization

Let us consider the objective function f:X→R , which measures the quality of the coffee as the percentage of area without bubbles visible in the coffee foam. X denotes a bounded domain X=[0,60]×[40,100]×[10,14] . A point x∈X is expressed as x=(t,s,h) , where t [s] is mixing time, s [%] is mixing speed, h [cm] is water pouring height. The goal is to maximize the function f over the bounded domain, i.e., to find $\arg\max_{x\in X}f\left(x\right)$ in order to effectively optimize for microfoam. As f(x) is unknown, Bayesian Optimization (BO), recognized as one of the most efficient sampling algorithms for black-box functions (Nguyen, 2019), was selected as the optimization method. It is particularly effective when only a few parameters need to be optimized (Saar et al., 2018). With BO, a Gaussian Process GP prior is placed on f(x) :

$$f\left(x\right)∼GP\left(m\left(x\right),k\left(x,x^{\prime }\right)\right),$$

where m(x) is the mean function, in this case set to zero, and $k\left(x,x^{\prime }\right)$ is a covariance kernel being a Matern kernel with ν=2.5 .

At iteration n , n distinct points ${\left\{x^{\left(i\right)}\right\}}_{i=1}^{n}\subset X$ have been observed, with corresponding values ${\left\{y^{\left(i\right)}\right\}}_{i=1}^{n}$ , where $y^{\left(i\right)}=f\left(x^{\left(i\right)}\right)+\epsilon^{\left(i\right)}$ , with $\epsilon^{\left(i\right)}$ accounting for observational noise. Given these data, the posterior predictive distribution for any new point is a normal distribution:

$$f\left(x\right)|{\left\{x^{\left(i\right)},y^{\left(i\right)}\right\}}_{i=1}^{n}∼N\left(\mu_{n}\left(x\right),\sigma_{n}^{2}\left(x\right)\right),$$

where μ and σ respectively correspond to mean and variance, whose parameters are fitted to the data by maximizing the Gaussian Process’ log marginal likelihood after each3 observation.

To decide the next experimental point $x_{n+1}$ , an Upper Confidence Bound (UCB) acquisition function was selected:

$$\alpha_{n}\left(x\right)=\mu_{n}\left(x\right)+\kappa \sigma_{n}\left(x\right),$$

where κ is a parameter for controlling the exploration-exploitation tradeoff. Hence, at step n , the next point $x_{n+1}$ is chosen as:

$$x_{n+1}=\arg\underset{x\in X}{\max}\alpha_{n}\left(x\right)=\arg\underset{x\in X}{\max}\left[\mu_{n}\left(x\right)+\kappa \sigma_{n}\left(x\right)\right].$$

The value of κ=8 was chosen, reflecting the need to explore the design space before exploiting and finding the optimal solution.

The more observations provided to the optimizer, the more confident the algorithm becomes regarding its prediction of optimal parameters. Despite the efficiency of this approach, searching a design space that has three parameters that exhibit variance and stochastic nature of the results requires tens or hundreds of trials to form an accurate model that can also provide a solution with a good performance.

The specific choices of BO components were inspired by prior studies and practical recommendations in the related literature Hertel et al. (2020); Oliveira et al. (2019); Hao et al. (2019); Riche and Picheny (2021). For example, Matern kernel’s balance between smoothness and flexibility makes it a commonly used default in black-box optimization tasks. UCB acquisition function was selected for its tunable exploration-exploitation trade-off and ease of implementation. Trial-and-error tests were conducted using different values of the exploration parameter κ . Among the values tested, κ=8 provided a reasonable trade-off between exploration and convergence speed during early-stage experiments. Future work could investigate the sensitivity of optimization results with respect to the selected parameters and alternative acquisition strategies (e.g., Expected Improvement or Probability of Improvement) to further validate robustness.

## 4 Results

### 4.1 Coffee preparation and optimization

First, to demonstrate the coffee making process and evaluate its repeatability, four cappuccinos were automatically prepared with the same input conditions. The resulting coffees had a bubble coverage of 22.36 ± 0.32%. While the localization of the bubbles on the foam surface varied, there were clear similarities in the density and size of the bubbles present. Although the sample size was limited, this provided an estimate of the natural variance introduced by the system. All efforts were made to conduct the experiments under consistent environmental and setup conditions. However, minor fluctuations in, e.g., ambient lab temperature or humidity could have contributed to results variation. This highlights the need for larger-scale physical experiments and for the use of BO in the optimization processes, as it can handle this variability.

**FIGURE 8:**
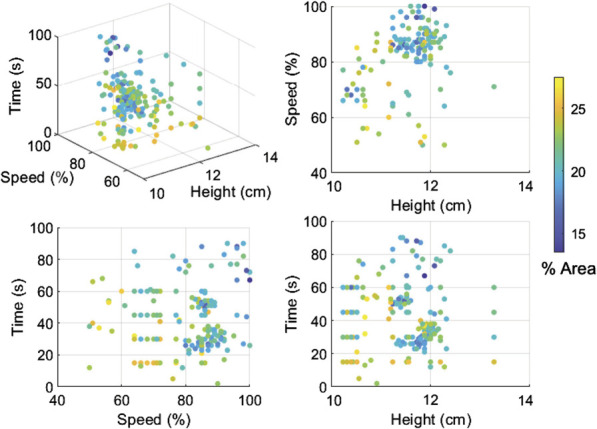
Bubble coverage results. The plots display data from over 100 coffees, prepared with varying stirring speed, stirring time and water pouring height.

Before performing BO, approximately 100 coffees were made using the experimental setup. This included a mix of grid-based and random exploration to investigate the design space and the observed variability. The bubble area of these coffees as a function of the mixing speed, time and pour height is shown in Figure 8. The results demonstrate the complexity of the interactions. It is evident that for the stirring speed lower than 60%–70% and mixing time below 20 s the coffee quality distinctly drops, resulting in high bubble coverage. With respect to the water pouring height, it is hard to indicate a clear trend–it may be due to the too narrow height range or low bubble area dependence on this parameter. Low pouring height and quick mixing at high speeds results in some instances of lowest bubble coverage. However, other combinations of parameters also produce high quality coffees. This further supports the use of BO, as there is no single local minimum that can be found with simple gradient descent-based methods.

**FIGURE 9:**
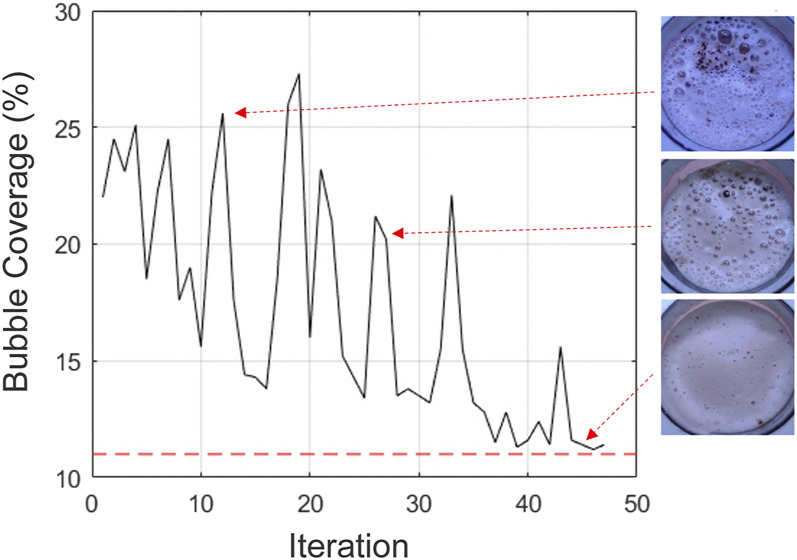
Bayesian Optimization for minimizing the bubble coverage. Although fluctuations are present in the optimization process, there is a convergence tendency.

**FIGURE 10:**
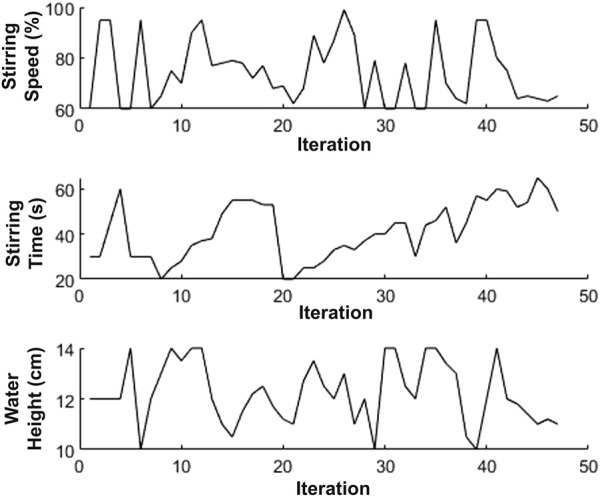
Parameter exploration in Bayesian Optimization. The proposed parameters values highly fluctuate, slowly converging from approximately 42nd iteration onwards.

Fifty coffees were prepared with the objective function of BO set to maximize the area without visible bubbles, which is equivalent to minimizing the bubble coverage. The results in Figure 9 show that although there are fluctuations in the optimization’s exploration, the process converges to a minimum value over time, with a minimal bubble area of around 11% found. This corresponds to a low water height (11 cm), low mixing speed (65%) and a high mixing time (50 s). From around 42nd iteration onwards, these values remain approximately constant with limited further exploration, particularly in the case of the mixing speed as presented in Figure 10.

**FIGURE 11:**
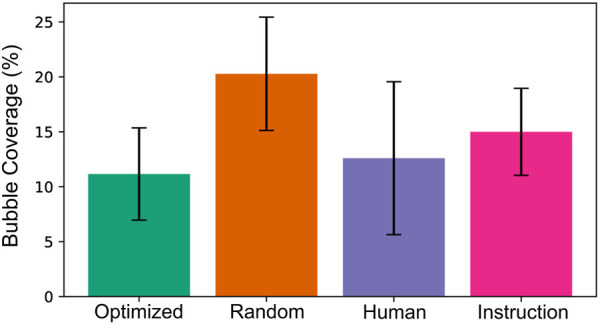
Comparison of the best results achieved by different methods. The plot presents the resulting average bubble coverage along with the standard deviation.

As BO provides no guarantee of optimality, the optimal processing conditions found were compared to others: 1) random selection of mixing conditions, 2) coffee preparation by a human, 3) robot following the provided producer’s instructions. For each of these cases, four coffees were made with the results presented in Figure 11, proving that BO-defined conditions outperformed the others. Although the human-made coffees had on average only one percentage point more bubble coverage compared to BO, they showed higher variance. Given that the human can use continuous visual feedback to adaptively mix the coffee, this highlights the quality of the optimal performance found using BO. The random preparation parameters presented the worst performance, and the instruction-based performance was inferior to the one of a human.

While this comparison provides a useful insight into the performance of the system, it should be noted that the number of repetitions in each group is limited. Therefore, the results should be interpreted as preliminary rather than statistically conclusive. Nevertheless, the observed trends support the method’s potential and motivate further investigation with larger sample size in future work.

**FIGURE 12:**
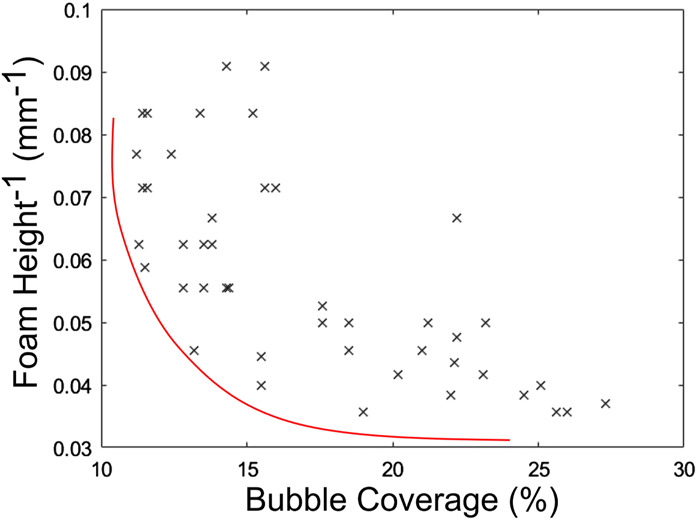
Quality metrics relationship. The subsequent increase in foam height and bubble coverage highlights a Pareto-optimal trade-off.

Additionally, the relationship between bubble coverage and foam height was investigated. For each of the 50 coffees prepared in the BO experiment, the foam height against the bubble coverage is plotted Figure 12. Interestingly, this showed that an increase in the bubble area is followed by an increase in the height of the foam. Therefore, there is a potential trade-off between foam height and bubble area, showing a Pareto-optimality problem. This requires further exploration in the future research.

### 4.2 Closed-loop clump removal

**FIGURE 13:**
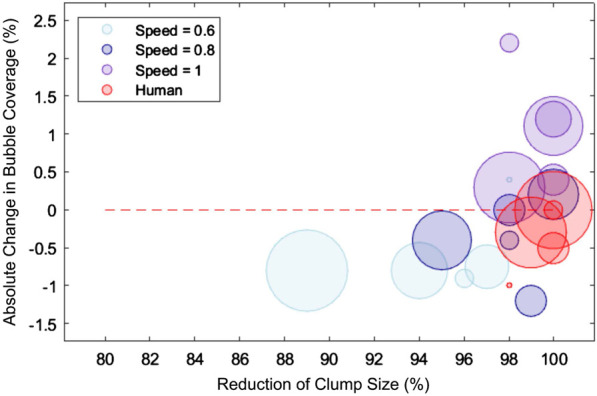
Results of the closed-loop clump reduction with the proportional controller. The plot illustrates the reduction in clump size and the absolute change in bubble coverage, with bubble radius in the plot representing clump size. The dotted line indicates zero change in bubble area.

This experiment examined the ability to detect and remove clumps, how this process affects the foam bubble area, and how similar the robot’s closed-loop removal is to human behavior. The coffees were prepared with the optimal parameters found with BO. The bubble area was recorded, and if there was a presence of clumps, their area was identified. The proportional controller was then used with varying mixing speeds ( 60% , 80% and 100% ). The results from the twenty experiments with the clump-containing cappuccinos are presented in Figure 13, showing the reduction in the clump size (i.e., the success of clump removal) and the change in bubble coverage area. For comparison, a human was asked to remove the clumps with a spoon, and the same metrics were recorded. As the occurrence of clumps is hard to reproduce on purpose, the clump size varied every time.

The ideal removal procedure should fully remove the clump and either reduce the bubble area or have minimal effect on it. Although high speed mixing removed the clumps, it had a negative impact on the foam by increasing the bubble coverage. Conversely, a low speed resulted in less success in removing the clumps, but reduced the bubble area. The speed of 80% of the maximum showed behavior most similar to that of humans, where the clumps were mostly reduced, but the change in bubble area is low. Interestingly, although the human was very good at removing the clumps, the bubble area did not decrease.

The results in this section demonstrate that similar behavior to humans can be achieved with the robotic setup. Additionally, the proportional controller proved to be a suited tool for the effective removal of clumps, even contributing to the improvement of foam quality.

## 5 Discussion

This work presented an automated approach to optimizing the foam of reconstituted beverages by leveraging robotics, computer vision, and optimization algorithms. The setup enabled systematic experiments under controlled conditions and demonstrated successful optimization of foam quality over 50 iterations. Another contribution of the work was the implementation of a computer vision-aided feedback loop that simulated human behavior in the removal of clumps and influenced the resulting properties of the foam.

While the focus was on foam optimization, the methodology highlights the broader potential of robotics and computer vision in food science, particularly for identifying optimal combinations of process parameters. This is increasingly relevant for developing products optimized for nutrition inclusion, cost, and processability. Compared to standard laboratory techniques for quality assessment, computer vision offered a non-contact, cost-effective, and versatile means of analyzing food and drinks.

The use of image-based algorithms to detect foam texture and clumps marks a step toward autonomous visual assessment. However, challenges remain in handling the stochastic nature of powdered beverages. Variability in ingredient proportion and foam formation - due to even slight changes in lab conditions - can affect optimization convergence, even when using identical input parameters. These aspects are similar to the challenges encountered in industrial food processing, where input materials naturally vary from batch to batch.

The results open several interesting directions for future investigation. Beyond foam, this study’s framework could be extended to optimize other food and beverage characteristics, such as color uniformity or foam stability. The current optimization considered only a limited number of parameters; expanding this set (e.g., with water temperature or stirrer geometry) could help capture more complex dependencies. Additionally, research on more advances fitness functions, such as combining foam quality with clump minimization, could extend the applicability of Bayesian Optimization to more real-world use cases. Finally, exploring alternative learning-based approaches, particularly those designed for image-based analysis, could offer a promising solution for generalizing the optimization approach. For example, a robot could be trained to reproduce the visual qualities of a provided food or drink sample.

In summary, this work demonstrates how robotics and computer vision can automate not only production steps, but also quality evaluation in food and beverage preparation. As food products become increasingly complex and consumer expectations rise, such technologies could support intelligent product development, assist in standardized preparation, and guide the creation of consumer instructions.

## Data Availability

The original contributions presented in the study are included in the article/supplementary material, further inquiries can be directed to the corresponding author.
